# A Novel Unsupervised Machine Learning-Based Method for Chatter Detection in the Milling of Thin-Walled Parts

**DOI:** 10.3390/s21175779

**Published:** 2021-08-27

**Authors:** Runqiong Wang, Qinghua Song, Zhanqiang Liu, Haifeng Ma, Munish Kumar Gupta, Zhaojun Liu

**Affiliations:** 1 Key Laboratory of High Efficiency and Clean Mechanical Manufacture, Ministry of Education, School of Mechanical Engineering, Shandong University, Jinan 250061, China; rqwang@mail.sdu.edu.cn (R.W.); melius@sdu.edu.cn (Z.L.); mahaifeng@sdu.edu.cn (H.M.); 2 National Demonstration Center for Experimental Mechanical Engineering Education, Shandong University, Jinan 250061, China; 3 Faculty of Mechanical Engineering, Opole University of Technology, 45-758 Opole, Poland; munishguptanit@gmail.com; 4 School of Information Science and Engineering, Shandong University, Qingdao 266237, China; zhaojunliu@sdu.edu.cn

**Keywords:** chatter detection, thin-walled parts, unsupervised machine learning, feature extraction, fractal theory

## Abstract

Data-driven chatter detection techniques avoid complex physical modeling and provide the basis for industrial applications of cutting process monitoring. Among them, feature extraction is the key step of chatter detection, which can compensate for the accuracy disadvantage of machine learning algorithms to some extent if the extracted features are highly correlated with the milling condition. However, the classification accuracy of the current feature extraction methods is not satisfactory, and a combination of multiple features is required to identify the chatter. This limits the development of unsupervised machine learning algorithms for chattering detection, which further affects the application in practical processing. In this paper, the fractal feature of the signal is extracted by structure function method (SFM) for the first time, which solves the problem that the features are easily affected by process parameters. Milling chatter is identified based on k-means algorithm, which avoids the complex process of training model, and the judgment method of milling chatter is also discussed. The proposed method can achieve 94.4% identification accuracy by using only one single signal feature, which is better than other feature extraction methods, and even better than some supervised machine learning algorithms. Moreover, experiments show that chatter will affect the distribution of cutting bending moment, and it is not reliable to monitor tool wear through the polar plot of the bending moment. This provides a theoretical basis for the application of unsupervised machine learning algorithms in chatter detection.

## 1. Introduction

In the cutting of thin-walled parts, chatter occurs easily due to the weak rigidity of the workpiece, which will greatly affect the machined surface quality, reduce production efficiency [1], accelerate tool wear [2], and even affect the fatigue life of the workpiece [3,4,5,6]. Therefore, effective methods to predict, control, and monitor chatter have been a focus of research in the past 20 years. There has been a relatively solid research base in chatter prediction, the stability lobe diagram (SLD) can be calculated by various methods, such as zero-order analytical (ZOA) [7], semi-discretization method (SDM) [8], full-discretization method (FDM) [9], differential quadrature method (DQM) [10], etc. Based on the research results in chatter prediction, chatter suppression has also made some important progress, such as active control methods [11,12,13] and passive damping control [14,15,16]. However, the dynamic characteristics of the system are constantly changing and easily disturbed [17]. As a result, the effectiveness of chatter suppression is easily affected by the accuracy of prediction methods. With the development of sensor technology and machine learning, the data-driven chatter monitoring method makes it possible to reproduce the machining process in real-time, which has become an intermediate to unify thin-walled parts cutting and chatter control.

In recent years, multi-sensor fusion technology has proved to be an effective way to monitor the cutting process [18], which is mainly because multi-sensor fusion reflects a variety of physical phenomena of the cutting process, covers multiple frequency ranges, and shares complementary information from different perspectives. As to chatter detection, with the help of wavelet transform, Tran et al. [19] identified milling chatter by the fusion of microphone and accelerometer sensors. Kuntoğlu et al. [20] compared the effectiveness of different sensors in the turning process and proved the high robustness of multi-sensor fusion method by acquiring cutting forces, vibration, acoustic emission, temperature, and current measurements. Since the multi-sensor fusion provides the possibility for high redundancy monitoring, its application in tool condition monitoring is also increasing. For example, Zhou et al. [21] monitored the condition of the cutting tool through a multi-sensor global feature extraction method during milling.

On the other hand, the development of machine learning technology makes the recognition of cutting conditions more convenient, which further promotes the practical application of the technology of milling process monitoring. Considering the advantage of identification accuracy, the supervised machine learning-based method is widely used in the monitoring of the cutting process. To avoid the influence of physical models, Denkena et al. [22] proposed a SLD calculation algorithm through support vector machines (SVM) and artificial neural network (ANN), and the highest classification accuracy of the proposed algorithm reaches 94%. Shi et al. [17] realized the early identification of chatter in high-speed milling by a kind of enhanced k-nearest neighbor method and a variety of extracted signal features. Wu et al. [23] proposed a tool wear identification method that does not require professional experience, which monitors the milling process based on the CNN model and the charge-coupled device (CCD) image sensor data. Thanks to the automatic detection algorithm, the mean absolute percentage error between the identified results and real tool wear value is only 4.76%. Sener et al. presented a chatter detection method based deep convolutional neural network (DCNN). It has been proven that the accuracy will be better if the process parameters are input to DCNN [24].

However, for cutting process monitoring, the supervised machine learning-based method has natural disadvantages, such as it must know the label of classification data in advance and need a complex process of training model. This limits the possibility of the practical application of condition identification, which is one of the main reasons why machine learning monitoring algorithms are difficult to be applied in commercial applications. Fortunately, some research on the application of unsupervised machine learning in cutting processes has emerged. Dun et al. [25] implemented the identification of milling chatter by autoencoder and hybrid clustering algorithm, and Peng et al. [26] implemented the monitoring of tool condition through extracting frequency-domain features of sound signals. However, the current methods mostly extract multiple features in the time domain, frequency domain, or time-frequency domain, and the recognition accuracy is still lower compared to supervised machine learning methods, which all lead to great inconvenience in their application to online monitoring. Therefore, an unsupervised machine learning method with convenient signal feature extraction and high recognition accuracy would be beneficial for the promotion of online monitoring for cutting conditions.

In fact, feature extraction is very important in pattern recognition. If the correlation between the extraction and the identification results is strong, even if the machine learning algorithm with low theoretical classification accuracy, such as unsupervised machine learning, can obtain better identification performance. The physical nature of the fractal feature, which describes the self-similar characteristics of signals, determines that the extracted feature is not easily influenced by the cutting process parameters, which provides a unique accuracy advantage for monitoring the nonlinear and non-stationary properties of chatter during the milling process [27]. At present, the method of extracting the fractal dimension of the signal is mostly based on box counting, such as Diykh et al. [28], who extracted the feature of fractal dimension of electronephhallgraphy (EEG) signals by box counting method, and classified the extracted datasets by combining support vector machine (SVM). Zhuo et al. also calculated the fractal dimension of the signals in the time-domain and frequency-domain separately by the box counting method for the identification of chatter in flank milling [29]. 

However, for the milling process of thin-walled parts, especially the thin-walled parts of hard-to-cut materials, due to the position dependence characteristics, tool wear coupling factors, and the time-varying dynamic characteristics of the milling system, the cutting signal has not only the characteristics of self-similarity but also self-affinity. The box counting method is also not suitable for solving the data with self-affinity [30]. On the other hand, the structure function method (SFM) has been widely used for the calculation of fractal dimension of rough surfaces because the self-affine characteristics of the surface profile can be considered in the process of constructing the function [31,32]. Therefore, it can be expected that the identification accuracy of cutting chatter can be improved by extracting features through the structure function method (SFM).

Given the problems of complex feature extraction and low identification accuracy in the current unsupervised machine learning-based cutting monitoring method, this paper first extracts the fractal features of signals by SFM and provides a novel chatter detection method based on fractal dimension and k-means clustering algorithm. The main advantage of the proposed method is that it can achieve the desired recognition accuracy by extracting only one kind of time-domain feature, which avoids the complicated process of combining multiple features extracted by traditional methods and improves the recognition accuracy of unsupervised machine learning-based methods. This provides a new idea for the research related to unsupervised machine learning-based cutting process monitoring. The rest of this paper is organized according to the following structure: Section 2 introduces the chatter identification method based on fractal dimension and k-means clustering algorithm. Section 3 describes the experimental design, feature extraction and experimental verification results in detail. Section 4 discusses the effective method of judging the chatter, and conclusion is drawn in Section 5.

## 2. Methodology

Commonly used time-domain features essentially describe the intensity and distribution characteristics of the signals. However, these physical features of signals are not necessarily related to the cutting conditions, and even changes in process parameters may affect such features. Considering the correlation between milling chatter and signal fractal features, the proposed method in this paper first extracts the time-domain features of the signal based on fractal theory, and then identifies the milling condition by data clustering. At the same time, three common time-domain features (root mean square, kurtosis, and skewness) are also extracted as the comparison to judge the accuracy of the proposed method.

### 2.1. Related Time-Domain Features

Since the subtle changes of the process will affect the parameters of signal features, the commonly used time-domain features have obvious advantages in identifying stationary signals. However, for nonlinear and non-stationary signals including cutting chatter, it is generally difficult to identify the state by time domain features alone.

Root mean square (RMS) is the most commonly used parameter in time-domain features, which is actually related to the total energy and reflects the strength of the milling signal. It has proven effective on many occasions. It can be expressed as:

(1) $$x_{rms}=\sqrt{\frac{1}{N}\sum_{i=1}^{N}x_{i}{}^{2}}$$

where *N* is the number of sampling points and represents the *i*th sampling point in the cutting signal $x_{i}$.

Kurtosis, on the other hand, expresses the characteristics of the numerical distribution of random variables, as shown in Equation (2), which is essentially a fourth-order normalized central moment of the data. It reflects the steepness of the distribution pattern of all samples. In terms of signal morphology, kurtosis represents the sharpness of the signal peak compared to the normal distribution, and the larger the kurtosis value, the greater the difference between the distribution of the data and the normal distribution.

(2) $$x_{kur}=\frac{1}{N}\sum_{i=1}^{N}{\left(\frac{x_{i}-\bar{x}}{\sigma_{i}}\right)}^{4}$$

where, $\bar{x}$ represents the average value of the signal data and $\sigma_{i}$ is the standard deviation of signal data.

The expression of skewness is shown in Equation (3), which is similar to kurtosis and also expresses the numerical distribution characteristics of random variables. However, differing to kurtosis, it is the third-order normalized central moment of the sampling data, which represents the symmetry of the distribution. The larger the absolute value of skewness, the more it deviates from the normal distribution.

(3) $$x_{skew}=\frac{1}{N}\sum_{i=1}^{N}{\left(\frac{x_{i}-\bar{x}}{\sigma_{i}}\right)}^{3}$$

It is generally believed that when chatter occurs during milling, the amplitude of the signal increases and the data distribution changes, which in turn leads to changes in kurtosis and skewness. This is the basic principle of identifying chatter by the time-domain features of the signal.

### 2.2. Fractal Dimension Based on SFM

The structure function method (SFM) is usually used to calculate the fractal dimension of the profile of rough surfaces, which is actually a set of data Z(x) associated with a sampling position x that satisfies [32,33].

(4) $$S(\tau )=\langle {\left[Z(x+\tau )-Z(x)\right]}^{2}\rangle =C\tau^{4-2D}$$

where S(τ) is defined as the structure function of the time-domain data, τ is the length of the random sampling point interval, and < > denotes the temporal average. Equation (4) elaborates the power-law relationship between the arithmetic average of the signal amplitude on different time scales and the fractal dimension. When 1 < *D* < 2, *C* is a constant, which is also related to the fractal parameter [34], and can be expressed as

(5) $$C=\frac{\Gamma \left(2D-3\right)\sin\left[\left(2D-3\right)\pi /2\right]}{2-D}G^{2\left(D-1\right)}$$

where *G* is the characteristic scale constant, and Γ represents the gamma function.

As for the signals of sound, acceleration, and cutting bending moment, the signal amplitude is actually composed of multiple discrete points according to the time series, i.e.,

(6) $$Z(x)=Z_{i}(i=0,1,2,\ldots ,N)$$

As a result, a random sample point interval length τ can be defined as

(7) τ=nΔt(n=1,2,3…)

For a given signal, the number of discrete points is related to the sampling frequency $f_{s}$ of the signal. Therefore, the distance between two discrete points satisfies $\Delta t=1/f_{s}$.

Then Equation (4) can be converted to

(8) S(τ)=S(nΔt)=〈[Zxi+nfs−Zxi]2〉=1N−n∑i=0N−nZi+n−Zi2 (i+n<N)

Taking the logarithm of both sides of Equation (8), it can be obtained that

(9) $$\mathrm{lg}S\left(\tau \right)=\mathrm{lg}C+\left(4-2D\right)\mathrm{lg}\tau$$

Therefore, the distribution of the structure function of the cutting signal in the double logarithmic coordinate system can be obtained by substituting Equations (6) and (7). So that in the double logarithmic coordinates (log-log coordinates), if $\mathrm{lg}S\left(\tau \right)$ and lgτ is linear and the slope 0 < α < 2, it can be determined that the signal data have fractal characteristics. The fractal dimension of the cutting signal can then be calculated:

(10) $$D=2-\frac{\alpha }{2}$$

### 2.3. Methodology of Chatter Detection

Once the signal features are extracted, the different milling conditions can be identified from the extracted features, which is also known as chatter detection. Here, to avoid the complicated process of model training, k-means clustering is used to achieve the identification of milling conditions. As a typical unsupervised machine learning method, it does not need to know the labels of the data in advance, but its clustering results are more sensitive to the number of divided classes. As for this paper, the extracted data can be classified into two classes, namely stable and chatter, which means the number of classifications is clear. This is a basic prerequisite to be able to apply the k-means clustering algorithm.

Assuming that the set of extracted original features is $\left(x_{1},x_{2},\ldots ,x_{n}\right)$, the number of classifications is *k* (*k* ≤ *n*), i.e., the original data is divided into *k* classes $S=\{S_{1},S_{2},\ldots ,S_{k}\}$, and the classification can be achieved by minimizing the following expression:

(11) $$\underset{S}{\arg\min}\sum_{i=1}^{k}\sum_{x_{j}\in S_{i}}{\left\|x_{j}-\mu_{i}\right\|}^{2}$$

where $\mu_{i}$ denotes the center vector (cluster center) of the classification *S_i_*. In order to solve this NP-hard problem, Equation (11) is mostly solved by multiple iterations. The derivation of Equation (11) yields that the objective function is minimized when $\mu_{i}$ is equal to the mean value of all the samples. That is

(12) $$\mu_{i}=\sum_{j=1}^{n_{i}}x_{j}/n_{i}$$

where, $n_{i}$ denotes the total number of samples in the *i*th cluster and $x_{j}$ is the *j*th sample in the *i*th cluster. Therefore, the basic principle of the method is to assume that data of the same class are closer, and thus to recognize the milling condition by calculating the distance of the data in the feature space.

**Figure 1:**
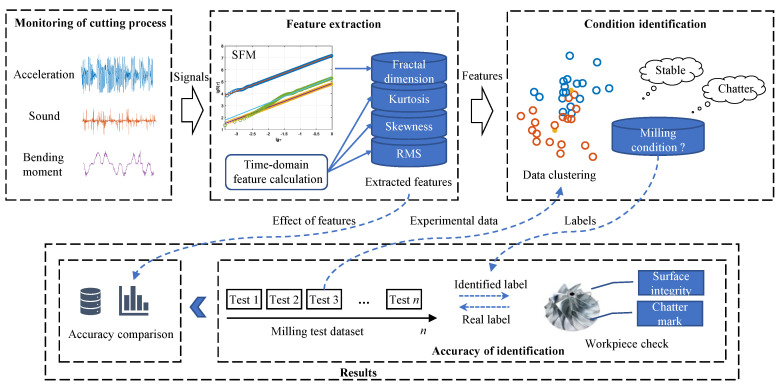
Proposed method.

As shown in Figure 1, the signals of acceleration, sound, and bending moment of the cutting process are acquired synchronously. The extracted multiple features are used as the three dimensions of the clustering data to fuse the multi-sensor information. Based on SFM, the fractal dimension of the multiple signals is then calculated. As a comparison, three kinds of commonly used time-domain features are also calculated. Further, the milling condition is identified by data clustering. In the process of pattern recognition, various signal features are clustered separately for accuracy comparison. Finally, the accuracy of the proposed method is verified by comparing the clustering output labels with the actual experimental milling condition, which is verified by various means such as workpiece inspection, FFT and cutting bending moment.

## 3. Results

### 3.1. Experimental Setup

**Figure 2:**
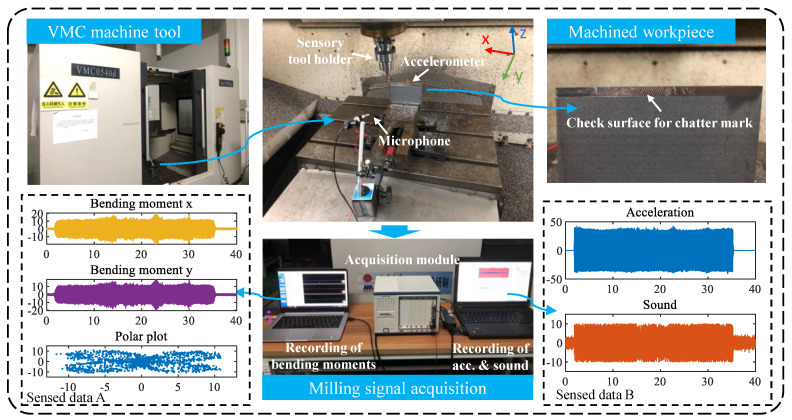
Experimental procedure.

The experimental procedure is shown in Figure 2. The milling experiments are performed on a VMC machine with a 4-tooth end mill of 10 mm diameter and a thin-walled titanium alloy part of 100 × 100 × 5 mm. The acceleration signals of the cutting process were collected by an accelerometer DYTRAN 3145A1 on the back of the workpiece to monitor the workpiece vibration. Acoustic signals are collected by a microphone (GRAS 46BE) near the tool and workpiece. The bending moment of the cutting process is collected by the sensory toolholder (SPIKE). The acceleration and sound signals are collected by the acquisition module (NI PXIE-4464) and transmitted to the PC recording system. The bending moment signals are collected by a read receiver unit and transmitted wirelessly to the PC. The polar plot of the cutting moment can then be obtained by the scatter distribution of the bending moment in both x and y directions.

Table 1 shows the design of the process parameters for the milling experiments. All experiments are performed with a cutting width of 0.2 mm, and each set of experiments is completed by observing the workpiece surface, performing FFT, and analysis of cutting bending moment to ensure that the corresponding cutting state is achieved, which will be described in detail in Section 3.3. It should be noted that only cutting chatter can be identified in this paper, and tool wear is not in the scope of this study. However, since the workpiece is titanium alloy, which is typically difficult to machine material, tool wear will inevitably occur during the actual cutting process. When the tool is worn, the amplitude of cutting force will change significantly and the surface quality of the workpiece will deteriorate, which makes the characterization of chattering more obvious. In fact, in order to clearly distinguish between the two signals, the tool under chatter conditions will inevitably be worn. How to identify cutting chatter and tool wear simultaneously will be the focus of our next research.

### 3.2. Feature Extraction

**Figure 3:**
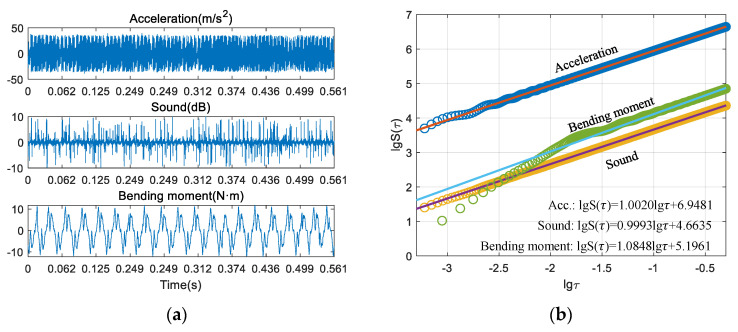
Calculation of the fractal dimension of the stable milling condition. (**a**) aquired signals; (**b**) process of calculating fractal dimension.

**Figure 4:**
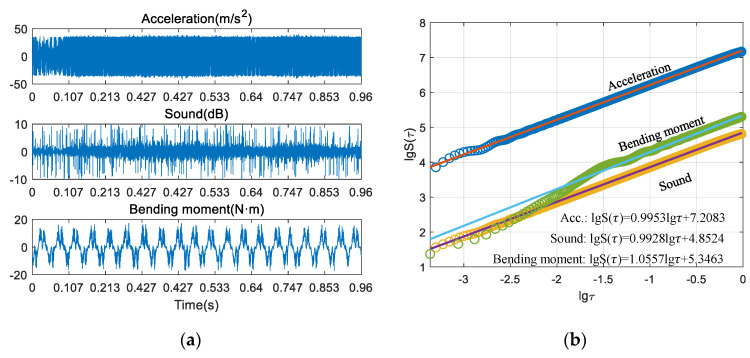
Calculation of the fractal dimension of the chatter condition. (**a**) aquired signals; (**b**) process of calculating fractal dimension.

Figure 3 and Figure 4 show the process of calculating the fractal dimension of multiple sensor signals, respectively. Figure 3a shows the cutting signals of one of the experimental tests in the stable condition, and Figure 3b shows the process of calculating the fractal dimension of each signal by the structure function method (SFM). Particularly, to avoid the influence of process parameters on the extracted signal features, the signal is segmented based on the spindle speed and sampling frequency [35]. Here, 20 revolution data are uniformly selected as the sampling signal. Since the spindle speed of each group of experiments is different, the time scale of each group of experiments is also inconsistent. The fractal dimension can be calculated by combining Equation (6) with the slope of the line obtained from the regression results. Figure 4 shows one of the cutting tests in the chatter condition. It can be seen through the time-domain data that the time-domain characteristics of these three signals are obviously different due to the different acquisition methods. Through Figure 3 and Figure 4, the acceleration, sound, and bending moment signals in both stable and chatter conditions have obvious scale-free intervals, indicating that each sensing signal of the milling process has fractal characteristics, and similar conclusions were obtained in references [27,29], which is the basis for applying fractal geometry to extract fractal features.

Moreover, from top to bottom, for acceleration, bending moment and sound, regardless of the stable or chatter conditions, the distribution patterns of the three kinds of signals in double logarithmic coordinates are almost the same. The scatter distribution pattern of each signal in the two different states is also similar. This is actually determined by the time-domain characteristics of the cutting signals. As shown in Figure 3a and Figure 4a, the time-domain characteristics of acceleration, sound, and cutting bending moment are obviously different due to the acquisition methods and the characterized physical quantities, but they all reflect the cutting process from different perspectives, which also shows the necessity of multi-sensor information fusion monitoring. Table 2 shows the final calculated fractal dimensions.

**Figure 5:**
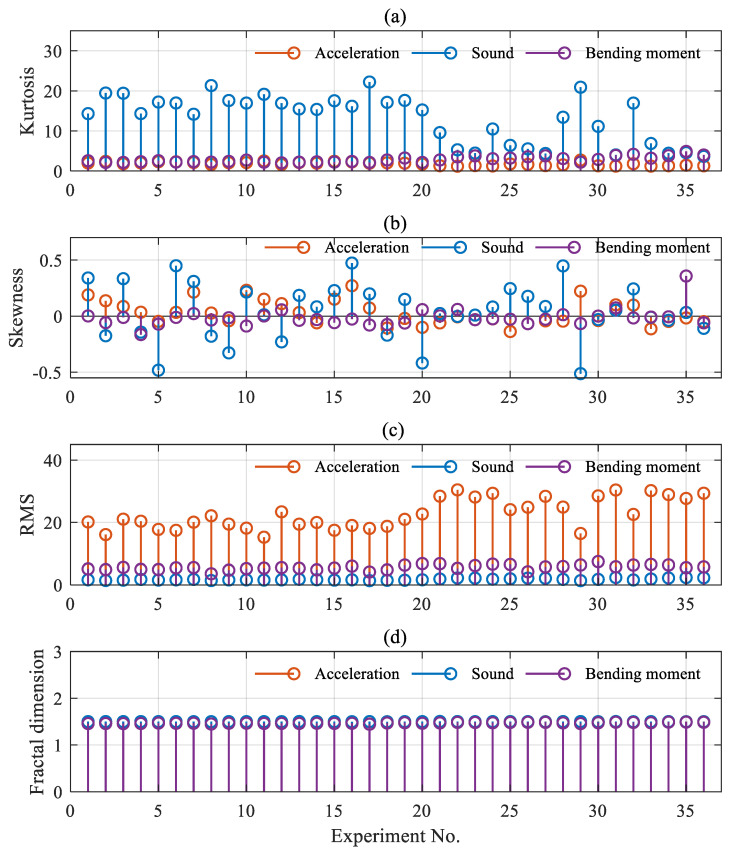
Distribution of features for different process parameters. (**a**) kurtosis; (**b**) skewness; (**c**) RMS; (**d**) fractal dimension.

In addition, as mentioned earlier, the amplitude and distribution of a given signal will change with the change of cutting parameters, which in turn leads to a change in the common time-domain characteristics. In other words, the common time-domain characteristics are susceptible to the process parameters. In general, the change of cutting process parameters may not be necessarily related to the cutting conditions, and the time-domain features can be easily affected by the process parameters, which ultimately makes it difficult to detect the cutting condition by a single time-domain feature. Figure 5 shows the distribution of each signal feature under different process parameters. The horizontal axis in the figure indicates the experiment serial number, which represents 36 groups of experiments, and it is obtained from 18 groups of experiments in Table 1, each group of which is taken as two segments. The variation of kurtosis, skewness, RMS, and fractal dimension of multi-sensor signals with the experimental sequence number are shown sequentially in Figure 5. And according to Table 1, different experimental serial numbers represent a variety of process parameters. As shown in Figure 5a–c, the conventional time-domain characteristics of both acceleration, sound, and cutting bending moment change significantly with the change of process parameters. But according to Figure 5d, the fractal dimensions of the three signals, when the process parameters are changed, remain at a certain level. This is mainly because the fractal dimension characterizes the self-similarity of the signals, and the change of the process parameters cannot change the degree of similarity of the signals.

### 3.3. Experimental Validation

The purpose of extracting signal features is to further identify the milling condition. Traditional supervised machine learning methods need to know the labels of the data in advance to train the machine learning model, and then recognize the corresponding conditions. However, the real milling condition cannot be known in advance during the actual machining process, so the identification of cutting chatter by supervised machine learning methods poses great difficulty to achieve online monitoring. As described in Section 2.3, to solve this problem, the k-means clustering algorithm is used in this paper to identify different milling conditions. The clustering algorithm, as a typical unsupervised machine learning method, does not need to foreknow the labels of the data, but its clustering results are more sensitive to the number of classified categories. In contrast, the milling condition monitoring involved in this paper mainly monitors milling chatter, that is, the extracted data can be classified into two states: stable and chatter. In other words, the number of clusters of the extracted data has been determined. This is the basic prerequisite to be able to apply the k-means clustering algorithm.

**Figure 6:**
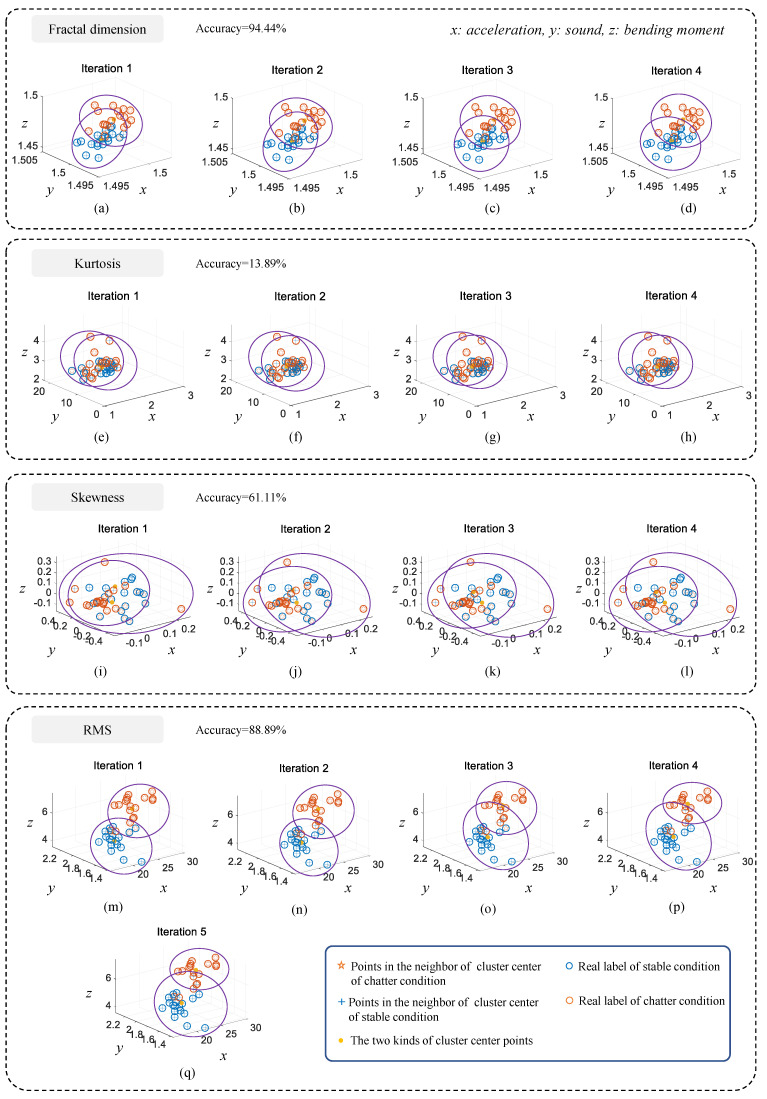
Iteration results.

In order to check the accuracy of different time-domain features for identifying milling chatter, all the extracted signal features are clustered separately in this section and the clustering results are examined according to the known labels. Moreover, the signal features of acceleration, sound, and cutting bending moment are taken as three dimensions of the feature space respectively, so as to complete the fusion of multi-sensing information. Note: In the process of applying the clustering algorithm, it is not necessary to know the data labels in advance, and the known data labels are introduced here to check the discrimination accuracy of different signal features. Figure 6 shows the clustering results for each signal feature. As shown in the figure, fractal dimension performs the best, achieving 94.44% classification accuracy after only four iterations, which is even better than some supervised machine learning methods.

In contrast, kurtosis performs the worst, with a final classification accuracy of only 13.89%, as shown in Figure 6e–h, mainly because there is no obvious distinction in its spatial distribution. Skewness and RMS have intermediate classification accuracies of 61.11% and 88.89%, respectively. Therefore, for monitoring milling chatter by different signal features, fractal dimension is the best, with a discrimination accuracy of 94.44%, skewness and RMS are less accurate and can basically identify chatter, while kurtosis clustering is the worst and cannot identify chatter from the extracted data. Furthermore, it is expected to obtain higher recognition accuracy if the fractal feature is combined with other time-domain features, frequency-domain features, and time-frequency-domain features. However, the purpose of this paper is to propose a time-domain feature that can be used alone, so as to improve the computational efficiency and apply it to on-line monitoring. Therefore, only a single time-domain feature is used for comparison in this paper. As for the combined effect of fractal features and other features, it is not only related to the feature extraction method, but is also affected by machine learning algorithm. The specific impact mechanism will be the content of our future research.

## 4. Discussion

As mentioned earlier, the clustering algorithm, as an unsupervised learning method, does not require prior knowledge of the data labels in identifying milling chatter. However, in this paper, in order to compare the discrimination accuracy of different signal features, definite labels are designed for each group of experiments, as in Table 1, the first nine groups of experiments are for stable conditions and the last nine groups of experiments are for chatter condition. However, whether the designed process parameters can indeed achieve the corresponding milling conditions still needs to be determined after the experiments. Indirect means are usually used to determine the occurrence of milling chatter, such as checking whether there are chatter marks on the surface of the workpiece, but judging the machining stability by surface quality is prone to error because the surface quality is easily affected by a variety of factors [25], which brings great uncertainty in tracking chatter by surface marks. Moreover, chatter is also not necessarily present throughout a process, thus judging based on the location of the chatter mark is not accurate. At the same time, it is difficult to detect the surface profile of the workpiece online. As a result, it is unrealistic to apply it in actual machining by stopping the machine after each cutting process to detect the surface quality.

**Figure 7:**
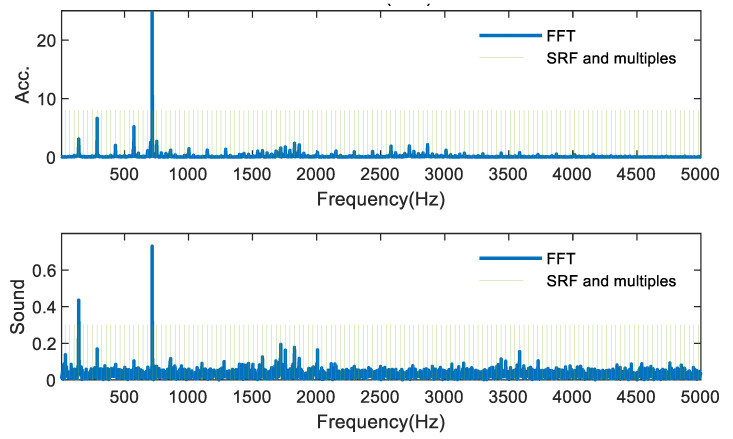
FFT for stable milling state experiments.

**Figure 8:**
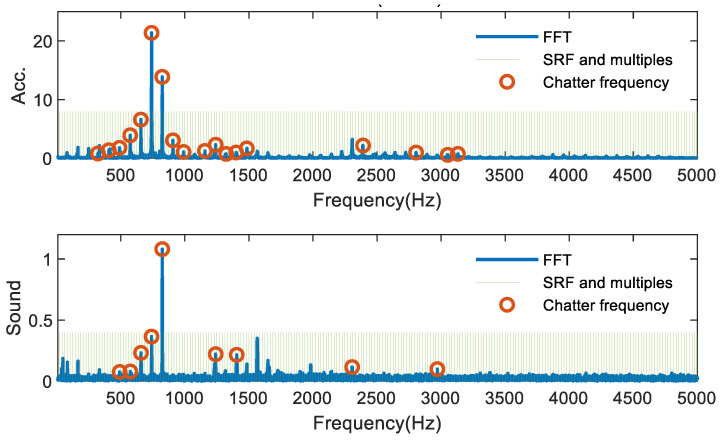
FFT for chattering milling condition.

Therefore, in actual experiments, the collected signals are mostly subjected to FFT to analyze the frequency components. When the signal spectrum contains only the tooth passage frequency, spindle rotation frequency and its harmonic frequency, the milling process is considered stable. On the contrary, it can be considered that milling chatter occurs if there are frequency components in the spectrum other than the tooth passage frequency, spindle rotation frequency and its harmonic frequency, and the extra frequency component is considered as the chatter frequency. Figure 7 shows the FFT of the acceleration and sound signals of one group of experiments in the stable milling condition, where the green vertical line represents the spindle rotation frequency and its multiplier frequency. The blue solid line represents the FFT result of the signal. In Figure 7, all of the signal spectra are the spindle rotation frequency and its multiplier frequency, which proves that the cutting process is in stable condition. As shown in Figure 8, the frequency components that are neither the spindle rotation frequency nor its multiplier frequency are the chatter frequencies, i.e., the part marked by red circles. It can be seen that the chatter frequencies are mostly set in the low and medium frequency parts, mainly because, in the thin-walled parts milling process, the chatter frequencies are mostly related to the natural frequency of the workpiece, which is mostly in the low and medium frequency band.

**Figure 9:**
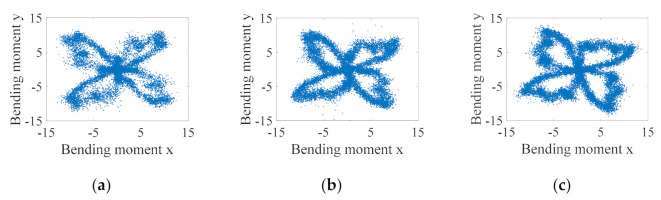

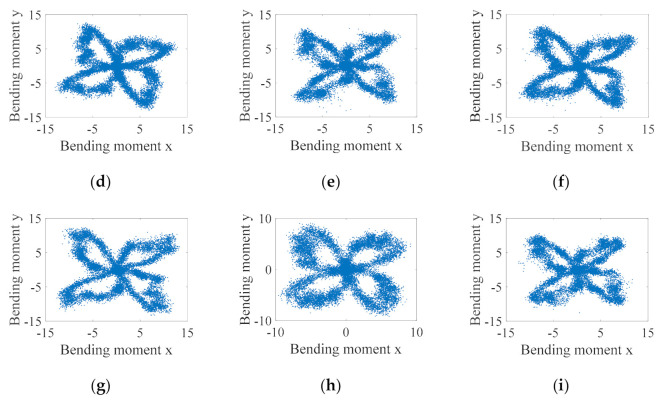
Polar plot of cutting bending moment for stable milling condition. (**a**) No. 1 experiment; (**b**) No. 2 experiment; (**c**) No. 3 experiment; (**d**) No. 4 experiment; (**e**) No. 5 experiment; (**f**) No. 6 experiment; (**g**) No. 7 experiment; (**h**) No. 8 experiment; (**i**) No. 9 experiment.

**Figure 10:**
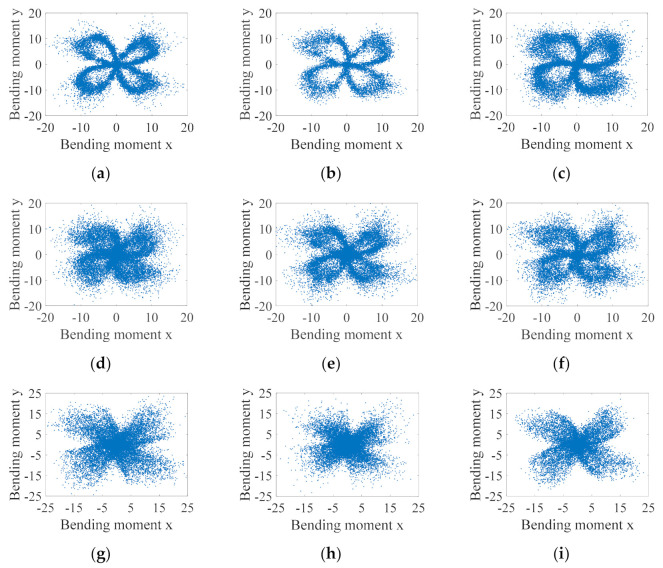
Polar plot of cutting bending moment for chatter milling condition. (**a**) No. 10 experiment; (**b**) No. 11 experiment; (**c**) No. 12 experiment; (**d**) No. 13 experiment; (**e**) No. 14 experiment; (**f**) No. 15 experiment; (**g**) No. 16 experiment; (**h**) No. 17 experiment; (**i**) No. 18 experiment.

Moreover, it is generally accepted that the cutting bending moment distribution can demonstrate the tool morphology to some extent, and it is often used in industrial software to monitor the tool condition. It was then found in this paper that the bending moment distribution does not necessarily correlate with tool condition alone, especially for thin-walled part milling, where milling chatter largely affects the tool morphology formed by the cutting bending moment distribution, which does not necessarily correlate with tool condition. Figure 9 shows the bending moment distribution for the stable cutting condition, i.e., the bending moment polar plot. Figure 9a–i represent nine groups of experiments for the stable milling condition, respectively. Benefiting from the stable contact between the tool and the workpiece, the bending moment distribution is more concentrated in the stable condition, and the tool morphology can be basically observed more obviously through the polar plot. In contrast, Figure 10 shows nine sets of experiments in the chatter condition, and it can be seen that the cutting bending moment distribution is relatively scattered due to the occurrence of chatter, and the tool morphology can hardly be determined. It is therefore not accurate to monitor the tool condition only by polar plots.

## 5. Conclusions

In order to improve the recognition accuracy of time-domain features for milling chatter, this paper applies the structure function method (SFM) for the first time to extract the fractal dimension of the signal as the time-domain feature, which avoids the influence of process parameters on identification accuracy. And by applying the k-mean clustering algorithm, it is not necessary to know the data labels in advance before identifying the milling condition, which eliminates the process of training models. The proposed method is more applicable to the actual cutting process monitoring. In this paper, multiple signal features are compared through the same learning algorithm, which verifies the accuracy of different signal features through experiments. However, the over fitting and generalization ability of the model are not only related to the extracted signal features, but also affected by the learning algorithm. The purpose of this paper is to provide a chatter detection method that is computationally efficient and conducive to improving the identification accuracy, so the research topic of this paper focuses on the influence of the extracted signal features. The following conclusions can be obtained from the whole paper.

1. Unlike traditional chatter monitoring methods that require extraction of multiple signal features, the proposed monitoring method uses only one kind of time-domain feature and requires basically no prior knowledge in either signal feature extraction or condition recognition, which improves computational efficiency and avoids the complex process of extracting multiple signal features. Due to improved computational efficiency, the proposed method can be a research basis for online monitoring of chatter during the milling of thin-walled parts.

2. In this paper, multiple signal features are extracted and clustered separately, and the accuracy of identification for milling chatter is compared. The results show that the fractal dimensional clustering is the best, with 94.4% accuracy using only a single time-domain feature, which is even better than some supervised machine learning methods. If combined with other time-domain features, frequency-domain features, and time-frequency-domain features, it is expected to obtain higher recognition accuracy, which can help promote the application of unsupervised machine learning in milling chatter monitoring.

3. For the monitoring of chatter by the clustering algorithm, it is difficult to achieve accurate identification of milling conditions by using other time-domain features alone. According to the results of the experiments, the best recognition accuracy among the traditional time-domain features is the root mean square, which reaches 88.89%, but it requires more iterations compared with other features, which means more computation. Although the number of iterations of skewness and kurtosis is less, their recognition accuracy is only 61.11% and 13.89%, respectively. It is obvious that it is difficult to accurately recognize chatter by these two time-domain features alone.

4. It is generally believed that the cutting bending moment distribution can show the tool morphology to a certain extent, and some existing commercial products also monitor the tool wear process by the polar plot. However, according to the experimental results in this paper, milling chatter can greatly affect the bending moment distribution, which indicates that monitoring the tool condition by polar plots is not reliable.

## Figures and Tables

**Table 1:** Process parameters for stable milling condition.

Millingn Condition	No.	Spindle Speed *s* (r/min)	$\mathrm{Cutting}\;\mathrm{Speed}\;v_{c}\;(m/\min)$	$\mathrm{Feed}\;\mathrm{Rate}\;v_{f}\;(\mathrm{mm}/\min)$	$\mathrm{Cutting}\;\mathrm{Depth}\;a_{p}\;(\mathrm{mm})$
Stable	1	2150	67.51	100	3
	2	2150	67.51	110	4
	3	2150	67.51	120	5
	4	2250	70.65	100	5
	5	2250	70.65	110	3
	6	2250	70.65	120	4
	7	2350	73.79	100	4
	8	2350	73.79	110	5
	9	2350	73.79	120	3
Chatter	10	1250	39.25	200	3
	11	1250	39.25	225	4
	12	1250	39.25	250	5
	13	1520	47.73	200	5
	14	1520	47.73	225	3
	15	1520	47.73	250	4
	16	1950	61.23	200	4
	17	1950	61.23	225	5
	18	1950	61.23	250	3

**Table 2:** Calculation results of fractal dimension.

Milling State	Sensing Type	Slope of the Regression	Fractal Dimension
Stable	Acceleration	1.0020	1.4990
Stable	Sound	0.9993	1.5004
Stable	Bending moment	1.0848	1.4576
Chatter	Acceleration	0.9953	1.5023
Chatter	Sound	0.9928	1.5036
Chatter	Bending moment	1.0557	1.4722

## Data Availability

The authors exclude this statement.
